# The Open Burning of Plastic Wastes is an Urgent Global Health Issue

**DOI:** 10.5334/aogh.4232

**Published:** 2024-01-12

**Authors:** Gauri Pathak, Mark Nichter, Anita Hardon, Eileen Moyer

**Affiliations:** 1 Aarhus University, DK; 2 The University of Arizona, USA; 3 Wageningen University and Research, NL; 4 University of Amsterdam and Amsterdam Institute for Global Health and Development, NL

**Keywords:** Plastic Pollution, Waste, Air Pollution, Plastic Burning

## Abstract

The open burning of mixed wastes that contain plastics is a widespread practice across the globe, resulting in the release of gas emissions and ash residues that have toxic effects on human and environmental health. Although plastic pollution is under scrutiny as a pressing environmental concern, it is often conflated with plastic litter, and the contribution of the open burning of plastics to air, soil, and water pollution gets overlooked. Therefore, campaigns to raise awareness about plastic pollution often end up leading to increased open burning. Many countries or regions where open burning is prevalent have laws in place against the practice, but these are seldom effective. In this viewpoint, we direct attention to this critical but largely overlooked dimension of plastic pollution as an urgent global health issue. We also advocate interventions to raise awareness about the risks of open burning and emphasize the necessity of phasing out some particularly pernicious plastics in high-churn, single-use consumer applications.

## Introduction

Plastic pollution has emerged as one of our most pressing environmental challenges, and media coverage of plastic pollution has skyrocketed. The public has encountered two differing narratives in the in the media. The first narrative unfolds with images of wildlife dying after ingesting plastics, mountains of plastic trash on land, and plastics strewn in the ocean. The second narrative showcases images of clean-up campaigns and innovative plastic repurposing and recycling programs, creating the impression that substantial efforts to address the problem are being made. However, the impact of these remediation efforts is subject to debate. While these efforts reduce visible plastic waste in the immediate environment, raise awareness about our growing plastic footprint, and inspire hope, in practice, less than ten percent of plastic waste [1, 2] is recycled. The plastic problem is steadily intensifying and is further worsened by the practice of shipping post-consumer plastics collected in higher income countries to low- and middle-income countries (LMICs) under the guise of recycling. This practice exacerbates the existing challenges LMICs face with their own plastic waste [3,4,5].

Most news reporting about plastic pollution has focused on litter as an environmental hazard to animals [6]. In comparison, far less media attention has been dedicated to the methods by which plastics are disposed and their resulting health consequences. News stories about the plastic waste collected locally by communities seldom specify what happens to this waste. Through our multi-sited ethnographic fieldwork, we found that this waste is routinely burned in open fires. Ironically, anti-litter campaigns and initiatives raising awareness about plastic pollution often contribute to increased volumes of open burning, as individuals and communities burn the trash collected in clean-up activities [7].

## Open Burning of Wastes

Approximately two billion people across the globe receive no municipal solid waste collection [8]. Their wastes are usually buried or dumped on land or in waterways, and more commonly, they are subjected to open burning [9]. The amount of plastics that get burned are estimated to be as high as the quantity of plastics emitted into the land or sea [10]. The scale of open burning among LMICs is estimated to range from 40% to 65% of total municipal solid waste [11,12,13]. Open burning is a large source of air pollutants, especially reactive trace gases and particulate matter (PM). For example, in China, emissions of PM 10 micrometers or less in diameter (PM10) from open domestic waste burning are equivalent to 22% of China’s total reported anthropogenic PM10 emissions [13]. When it comes to open burning, plastics are a particularly problematic waste stream. One study attributed 90% of black carbon emitted from burning wastes to two plastics—polyethylene terephthalate and polystyrene [14]. Although reliable measurements of the amount of black carbon released through the burning of wastes are lacking, very basic calculations have suggested that they are not negligible [10, 15], correlating the open burning of wastes to climate change as well as detrimental health effects. Some emissions, including persistent organic pollutants such as polycyclic aromatic hydrocarbons and dioxins and dioxin-related compounds, have been linked to skin lesions, cancer, immunological issues, and birth defects, among other health issues [11, 14]. The open burning of plastics is also associated with increased risks of heart disease, respiratory issues, and neurological disorders [7, 11, 12, 16,17,18]. The ash from open burning can contain dioxins, heavy metals, and other toxicants, which once settled on the ground, contaminate the soil, groundwater, and thus the organisms surrounding the environment and their respective food chains [19]. One study estimated a global mortality rate between 400,000–1,000,000 people per annum associated with waste mismanagement and suggested that plastic waste is likely to be responsible for a significant proportion of these deaths [20].

Open burning is a common practice in much of the Global South, and it occurs even in the Global North. With the trends of increasing global plastic production and consumption [9], it is likely that open burning will increase in frequency and extent. Our ethnographic fieldwork in countries such as India, Indonesia, the Philippines, and Zambia reveals a widespread reliance on open burning, despite the implementation of laws against the practice.

Individual consumers and communities cannot be blamed for open burning practices. We found that trash collection is infrequent or non-existent in many neighborhoods, and if available, can be an economic burden for households. Often, collected wastes are transported to dumping grounds burdened beyond their capacities. Simultaneously, low-value post-consumer plastic wastes from high-income countries are shipped to LMICs under the guise of recycling, even though these wastes are not always recycled. Post-consumer plastic wastes are often discarded along roadsides, in vacant lots, or in open dumps that are quickly overrun. In all such situations, when plastic wastes accumulate beyond the point of being deemed a nuisance—whether in terms of contagion or aesthetics—they are burned in the open.

## Health Consequences

The health consequences of open burning depend on the type of plastic being burned. Some plastics release particularly carcinogenic or toxic gases that pose significant health risks when burned (see Table 1) [7, 11, 13, 16]. Our fieldwork revealed that few people realize the magnitude of the health and environmental hazards of burning plastics, even plastics such as polyvinyl chloride or Styrofoam (a type of polystyrene) that release toxic dioxins, chlorinated furans, or styrene gas when burned in the open. The major concern voiced by interlocutors was the acrid smell and thick smoke from the burning of some plastics; this was considered more of a short-term inconvenience than a long-term hazard. However, the toxicants released by burning plastics can linger even after the smoke and smell have dissipated. In addition, the small-scale community burning of mixed plastic wastes has been found to pose greater risks to human health than fires at large dumping grounds because of the higher frequency, higher probability of human exposure, and low dispersive dilution caused by ground-level emissions [21].

**Table 1:** Examples of plastics that release toxicants upon burning.


TYPE OF PLASTIC	COMMON FORMS	TOXICANTS RELEASED UPON BURNING	HEALTH EFFECTS

Polyvinyl Chloride	Drainpipes, blister packs, children’s toys, bottles and jugs, etc.	Carbon monoxide, dioxins, chlorinated furans	Carcinogenic, birth defects, respiratory disorders, etc.

Polystyrene, styrene	Foam cups, meat trays, egg cartons, plastic forks and spoons, etc.	Styrene gas, acrolein, hydrogen cyanide	Carcinogenic, eye and mucous membrane damage, narcosis, death in high doses

Polyurethane	Wood finishes, sealants, adhesives, curtains, etc.	Carbon monoxide, hydrogen cyanide, phosgene	Death in high doses


Pollution has been deemed responsible for 15% of all deaths and 275 million disability-adjusted life years in 2017 [22]. The open burning of plastic wastes is an important contributor to such pollution. It is also an often-missed element, i.e the open burning of domestic wastes is an emissions source that has not been built into mainstream emissions inventories [13, 23, 24]. Laws against open burning exist but have not proven effective given a lack of alternatives for safe and convenient disposal, the shipping of low-value plastic wastes to LMICs, and low awareness regarding the health and environmental risks of open burning [7]. Furthermore, as mentioned earlier, anti-plastic pollution campaigns focusing on litter have paradoxically *increased* open burning as communities engage in clean-up activities and burn the collected waste.

## Recommendations

Plastic pollution has been recognized in public discourse as an urgent environmental challenge, and there are indications that governments are under pressure to take action to address the problem. Consequently, a UN Treaty on plastic pollution is currently being negotiated. It is imperative that any global plastics treaty recognizes the open burning of plastic wastes as a key aspect of plastic pollution and an immediate public health concern. Despite public discourses focusing on the associated health risks posed by microplastics and the endocrine-disrupting chemicals used as additives in plastics —both of which are subject to ongoing research— the open burning of plastic wastes should remain a paramount topic to address.

Interventions to address the open burning of mixed wastes containing plastics should not solely focus on community outreach programs to raise awareness of the associated risks. To increase efficacy, these interventions must also involve waste pickers and the informal recycling sector. Easily comprehensible labeling should be implemented to identify the types of consumer plastics that are particularly pernicious when burned, going beyond existing resin recycling codes. Crucially, the onus of collecting and safely disposing of those plastics should be placed on the plastic industry and consumer goods companies rather than on the state. At the same time, prioritizing the phase-out of the production of these particularly harmful types of plastics (e.g., polyvinyl chloride, Styrofoam) for short-term and high-churn applications is of vital importance. The implementation of effective policies necessitates pragmatic solutions based on systematic analysis in local worlds and cooperation not just between various national ministries of environment but also with the better-funded and agenda setting ministries of health. Furthermore, it will require the re-evaluation of the burden of responsibility for consumer plastic disposal, shifting it away from individual consumers and communities and more towards the plastic manufacturers and the consumer brand owners using plastic packaging.
